# Evaluation of Right Ventricular Function and Dyssynchrony in a Dog Model of Acute Pulmonary Embolism: Diagnostic Utility and Reversibility

**DOI:** 10.3389/fvets.2022.861064

**Published:** 2022-06-20

**Authors:** Tomoya Morita, Kensuke Nakamura, Tatsuyuki Osuga, Sei Kawamoto, Shingo Miki, Mitsuyoshi Takiguchi

**Affiliations:** ^1^Laboratory of Veterinary Internal Medicine, Department of Veterinary Clinical Sciences, Graduate School of Veterinary Medicine, Hokkaido University, Sapporo, Japan; ^2^Laboratory of Veterinary Internal Medicine, Department of Veterinary Sciences, Faculty of Agriculture, University of Miyazaki, Miyazaki, Japan

**Keywords:** pulmonary hypertension, microsphere injection, speckle tracking echocardiography, Tei index, cardiac catheterization

## Abstract

**Background:**

The diagnosis of acute pulmonary thromboembolism is challenging in dogs. Little has been reported on changes in echocardiographic indices in dogs with acute pulmonary thromboembolism. The objective of this study was to validate the relationship between echocardiographic indices and right heart catheterization variables in dogs with acute pulmonary embolism and to identify a useful echocardiographic index for diagnosing acute pulmonary embolism.

**Materials and Methods:**

Six healthy laboratory beagles were included in the study. Echocardiography and right heart catheterization were performed in a dog model of acute pulmonary embolism produced by microsphere injection. Echocardiographic indices, including the right ventricular (RV) Tei index, RV longitudinal strain, and the dyssynchrony index using speckle tracking echocardiography, transmitral flow, and eccentricity index, were measured.

**Results:**

The mean pulmonary arterial pressure increased (22.2 ± 1.2 mmHg) and the blood pressure decreased after microsphere injection. Although the mean pulmonary arterial pressure remained elevated, the blood pressure recovered 2 days after the microsphere injection. Most echocardiographic indices of RV function were significantly impaired following microsphere injection and recovered after 2 days. In contrast, the RV Tei index was significantly impaired after microsphere injection and the impairment persisted after 2 days. Multivariable analysis revealed that the RV Tei index was an independent echocardiographic predictor of pulmonary vascular resistance (β = 0.88, *P* < 0.001), and transmitral early diastolic wave was an independent predictor of the cardiac index (β = −0.86, *P* = 0.001).

**Conclusions:**

The RV Tei index is a useful echocardiographic index for diagnosing acute pulmonary embolism. Ventricular interdependence may be an important factor causing low cardiac output in dogs with acute pulmonary embolism.

## Introduction

The right ventricle is vulnerable to acute increases in afterload (pressure overload), resulting in a rapid decline in stroke volume despite good adaptation to preload (1). Acute pulmonary thromboembolism (PTE), a typical disease of acute right ventricular (RV) pressure overload, is a life-threatening condition that leads to respiratory distress, lethargy, pleural effusion, and ascites due to RV dysfunction, hypotension, cardiogenic shock, and ultimately death (2). Accurate diagnosis of acute PTE is important to relieve clinical signs and improve prognosis. In 1999, Johnson et al. reported that over a 10-year period in the United States, 0.9% of dogs were diagnosed with PTE at postmortem (2). In human medicine, computed tomography has a high sensitivity and specificity for the diagnosis of acute PTE (3). Because computed tomography has become increasingly popular in veterinary medicine, its accuracy of PTE diagnosis in dogs may improve (4). However, computed tomography is not widely performed in dogs with respiratory distress or hemodynamic instability because it is time consuming and requires general anesthesia.

Echocardiography may be another useful diagnostic tool for acute PTE because it is non-invasive, provides real-time data, and does not require anesthesia. Impaired echocardiographic indices and reversibility of echocardiographic indices in human patients with acute PTE have been reported (5–11). However, little is known about echocardiographic findings in dogs with acute PTE. One study in dogs demonstrated that only 5 of 29 dogs underwent echocardiography, two dogs presented with RV and pulmonary arterial dilation, and only one dog had pulmonary hypertension (PH) (2). In addition, there is no study performing both echocardiography and right heart catheterization in dogs. Therefore, the utility of echocardiography in the diagnosis of acute PTE in dogs is unknown. Clinical studies cannot reveal the relationship between echocardiographic findings and hemodynamics because right heart catheterization is difficult to perform. Therefore, an experimental study using a dog model of acute pulmonary embolism is required to clarify the utility of echocardiography as a diagnostic tool.

We previously reported the effects of acute RV pressure overload on echocardiographic indices of RV function and dyssynchrony in dogs with mild RV pressure overload induced by continuous intravenous infusion of U46619, a thromboxane A_2_ analog (12). In that study, RV Tei index, RV longitudinal strain (RVLS), and dyssynchrony index determined by 2D speckle-tracking echocardiography were independent predictors of mean pulmonary arterial pressure (PAP), pulmonary vascular resistance (PVR), and cardiac index (CI). Therefore, these echocardiographic indices were useful for assessing hemodynamic changes under mild RV pressure overload. However, the dog models using U46619 increased PAP and PVR as well as arterial blood pressure, pulmonary arterial wedge pressure, and systemic vascular resistance (SVR) resulting from systemic vascular constriction. In view of this, our previous findings could not be directly extrapolated to dogs with acute PTE. Additionally, time-dependent changes (e.g., acute period vs. compensation period) of echocardiographic indices under acute RV pressure overload are also unknown.

Accordingly, in this study, we evaluated the effects of acute RV pressure overload on echocardiographic indices of RV function and dyssynchrony in a dog model of acute pulmonary embolism in the acute and compensation periods.

## Materials and Methods

### Animals

Six male laboratory beagles (age, 1–3 years; weight, 10.8–12.8 kg) were used in this study. Complete physical examinations, electrocardiography, and standard echocardiographic examinations revealed that all dogs were healthy and possessed normal heart anatomy and myocardial function. All procedures were approved by the Laboratory Animal Experimentation Committee of the Graduate School of Veterinary Medicine, Hokkaido University (Approval No. 15-0087).

### Establishment of a Dog Model of Acute Pulmonary Embolism

The dogs were anesthetized with intravenous propofol (6 mg/kg) and intubated. The dogs were then administered heparin sodium (100 IU/kg, IV), cefazolin sodium hydrate (20 mg/kg, IV), and atropine sulfate (0.05 mg/kg,). Anesthesia was maintained using a mixture of 1.5–2.0% isoflurane and 100% oxygen. The end-tidal partial pressure of carbon dioxide was monitored and maintained between 35 and 45 mmHg using mechanical ventilation. The tidal volume was 10–15 mL/kg and the respiratory rate was maintained at 10–12 breaths/min. The anesthetized dogs were positioned in right lateral recumbency. An implantable port device (Bard Slimport; Medicon Ltd., Osaka, Japan) was percutaneously inserted into the left jugular vein, and the catheter tip of the device was inserted into the cranial vena cava. The port was affixed to the subcutaneous tissues on the left side of the neck. After surgery, buprenorphine (0.02 mg/kg, SC, twice daily) was administered as needed for analgesia, and cephalexin (20 mg/kg, PO, twice daily for 7 days) was used to prevent bacterial infection.

One week after port device implantation, the dogs were anesthetized using the same protocol. The dogs were positioned in left lateral recumbency. Heart rate and arterial blood pressure *via* arterial catheterization were monitored and recorded using a polygraph instrument (RMC-4000; Nihon Kohden Co., Tokyo, Japan). Lactated Ringer's solution was infused at a rate of 5 mL/kg/h *via* the cephalic vein.

Following a stabilization period of approximately 10 min, baseline recordings of hemodynamic variables and echocardiographic indices were performed. After the baseline measurements were obtained, acute pulmonary embolism was induced by the injection of 300-μm microspheres (Sephadex G50 Coarse; GE Healthcare UK Ltd., Amersham Place, Buckinghamshire, England) into the pulmonary artery *via* the port device. The volume of microspheres injected in each dog was adjusted to increase systolic PAP by 10–15 mmHg as assessed *via* right heart catheterization. Fifteen minutes after microsphere injection, hemodynamic variables were measured, and echocardiography was performed (acute period). Following the examinations, the dogs were allowed to recover from anesthesia. The dogs were monitored clinical signs twice a day by a veterinarian. None of treatment was needed. Throughout this study protocol, none of the dogs showed any clinical symptoms associated with pulmonary embolism, including dyspnea, syncope, dyspnea, lethargy, and ascites. None of the dogs died in this study. After 2 days, hemodynamic variables and echocardiographic indices were measured with the dogs under general anesthesia (compensation period). After compensation period, these dogs were repeatedly injected to create a dog model of chronic pulmonary embolism (13).

### Hemodynamic Measurements

All hemodynamic variables were recorded using a polygraph. A 6-Fr introducer sheath (Fast-Cath hemostasis introducers; St. Jude Medical Inc., Minnetonka, MN, USA) was percutaneously inserted through the right jugular vein, and a 5-Fr Swan-Ganz catheter (Swan-Ganz thermodilution catheter; Edwards Lifesciences Corp., Irvine, CA, USA) was inserted and advanced into the pulmonary artery under fluoroscopic guidance. The systolic, mean, and diastolic PAP, mean RV pressure, mean central venous pressure, and mean pulmonary arterial wedge pressure were measured and calculated as the average of five consecutive cardiac cycles. The cardiac output was measured using the thermodilution technique and calculated as the average of four measurements. The cardiac index (CI) was calculated by dividing the cardiac output by the body surface area. The first derivative of the maximum RV pressure change (max dP/dt) was calculated using the RV pressure data. The PVR, SVR, and PVR to SVR ratios were derived as follows:


$$\begin{array}{l} PVR\left(WU\right)\\ =\frac{\left(mean\;PAP-mean\;pulmonary\;arterial\;wedge\;pressure\right)}{cardiac\;output}\\ SVR\left(WU\right)\\ =\;\frac{\left(mean\;blood\;pressure-mean\;central\;venous\;pressure\right)}{cardiac\;output} \end{array}$$

### Echocardiographic Measurements

Echocardiographic examinations were performed by an echocardiographer (KN), using Artida (Toshiba Medical Systems Corp., Utsunomiya, Tochigi, Japan) equipped with a 3–7 MHz sector probe (PST-50BT; Toshiba Medical Systems Corporation, Utsunomiya, Tochigi, Japan) and HI VISION Preirus (Hitachi Aloka Medical Ltd., Tokyo, Japan) equipped with a 3–6 MHz sector probe (EUP-S52, Hitachi Aloka Medical Ltd., Tokyo, Japan). The HI VISION Preirus was used to measure the RV Tei index using a dual pulsed-wave Doppler (DPD). All dogs were placed in the left lateral recumbent position and examined. An ECG trace (lead II) was recorded simultaneously with echocardiographic imaging, and the heart rate was automatically measured.

Early diastolic (E) and late diastolic wave of mitral inflow were determined using a pulsed-wave Doppler with an apical 4-chamber view. To evaluate the degree of interventricular septal flattening, the eccentricity index (EI) was measured as the ratio of the long-axis to short-axis diameters of the LV at end-diastole and end-systole with a right parasternal short-axis view at the level of the papillary muscles (14). The systolic tricuspid annular velocity (S'_TV_) was determined using tissue Doppler (TDI) at the lateral tricuspid annulus with an apical 4-chamber view. The tricuspid annular plane systolic excursion was obtained by placing an M-mode cursor over the tricuspid valve annulus with an apical 4-chamber view and measuring the amplitude of motion during systole. The RV end-diastolic and end-systolic areas were obtained by tracing the RV endocardium in systole and diastole from the annulus to the apex with an RV-focused apical 4-chamber view, which included the RV apex, and then the fractional area change (FAC) was calculated as follows:


$$\begin{array}{l} FAC=\frac{RV\;enddiastolic\;area-RV\;endsystolic\;area}{RV\;enddiastolic\;area}\times 100 \end{array}$$

**Figure 1:**
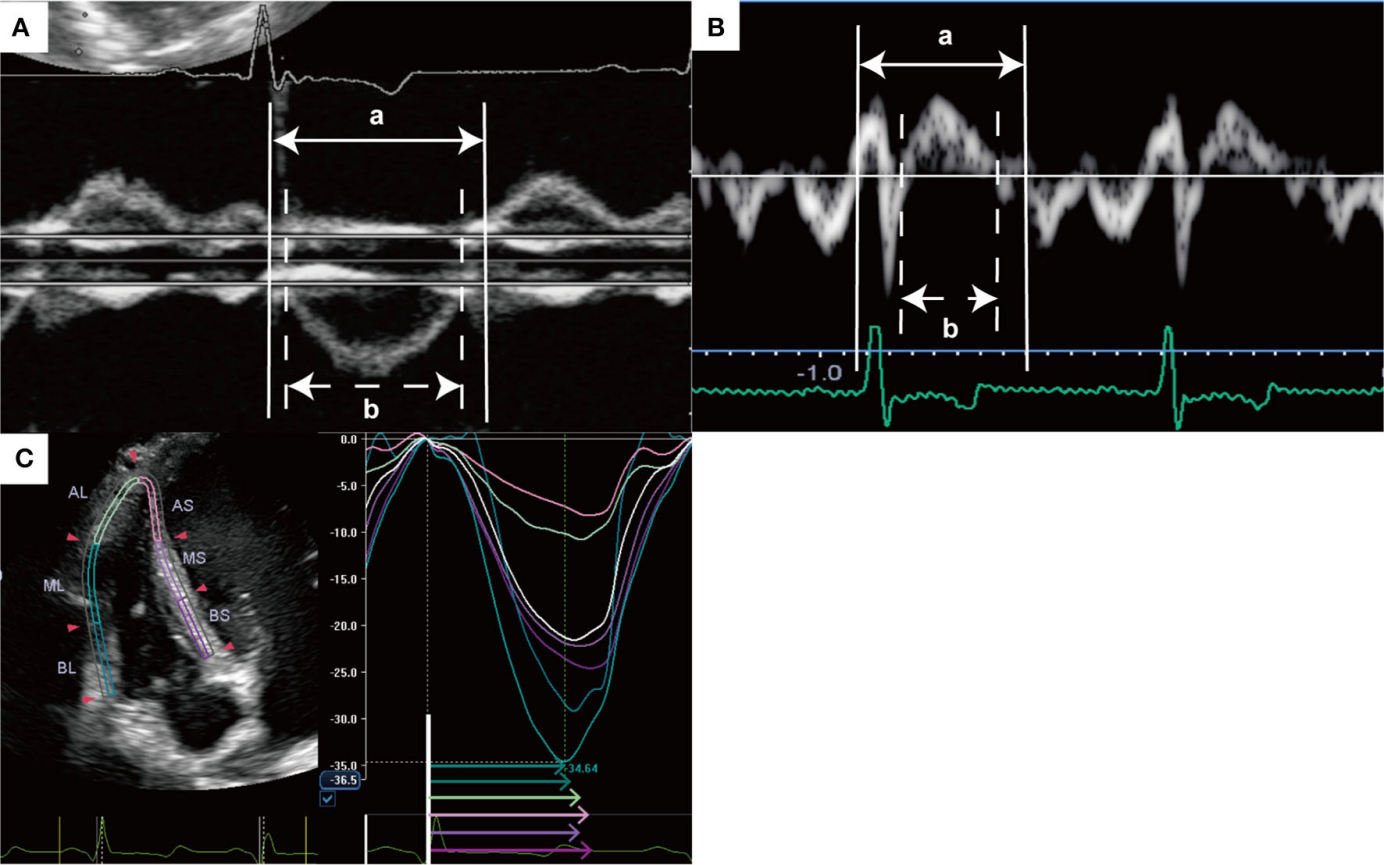
Representative measurement of right ventricular Tei index and speckle tracking echocardiography. **(A)** Right ventricular Tei index was measured using dual pulsed-wave Doppler with a left parasternal short-axis view as follows: (a-b)/b. **(B)** Right ventricular Tei index was measured using tissue Doppler with an apical 4-chamber view as follows: (a-b)/b. **(C)** Right ventricular longitudinal strain and RV-SD6 were measured by speckle tracking echocardiography with a RV-focused apical 4-chamber view. Right ventricular free wall and septum were automatically divided into three segments (apical, middle, and basal). Global RVLS was calculated by averaging the peak longitudinal strain values in all six segments of the RV, and free wall and septal RVLS were calculated by averaging each value of three segments. This image shows the global RVLS. RV-SD6 was calculated as the standard deviation of the systolic shortening time of six right ventricular segments. The colored arrows indicate segmental systolic shortening time. AL, apical lateral free wall; AS, apical septum; BL, basal lateral free wall; BS, basal septum, ML, middle lateral free wall; MS, middle lateral septum; RV-SD6, standard deviation of the systolic shortening time of right ventricular six segments.

The RV Tei index was calculated as the sum of the isovolumic time divided by the ejection time. The Tei index was calculated after image acquisition using DPD and TDI. The tricuspid inflow and pulmonary artery flow were measured simultaneously using DPD with a left parasternal short-axis view, and the sum of the isovolumic time was derived by subtracting the ejection time from the time of cessation of the late diastolic wave of the tricuspid valve to the onset of the E wave of the tricuspid valve in an image (15) (Figure 1A). To measure the RV Tei index by TDI, ejection time was defined as the duration of S'_TV_, and the sum of the isovolumic time was calculated by subtracting the duration of S'_TV_ from the interval from the end of the late diastolic tricuspid annulus velocity to the onset of the early diastolic tricuspid annulus velocity (16) (Figure 1B).

Speckle tracking echocardiography with an RV-focused apical 4-chamber view was performed, as previously described (17). Three consecutive cardiac cycles were stored on a hard drive for offline analysis with measurements made in offline software (2D wall motion tracking; Toshiba Medical Systems Corporation, Utsunomiya, Tochigi, Japan). The RV free wall and septal wall were each divided into three segments (basal, middle, and apical). The RVLS was obtained for each segment from the software-generated strain curves. The global RVLS was calculated by averaging the values observed in all six segments of the RV; free wall and septal RVLS were calculated by averaging each value of the three segments (Figure 1C). The systolic shortening time (SST) was defined as the time interval from QRS onset to peak longitudinal strain (Figure 1C). The free wall delay was calculated as the septal SST subtracted from the free-wall SST. The standard deviation of the SST of six RV segments (RV-SD6) was calculated to quantify RV dyssynchrony, and RV-SD6 was corrected for the R-R interval according to Bazett's formula (18).

### Statistical Analysis

Statistical analyses were performed using JMP statistical software (version 12.0; SAS Institute Inc., Cary, NC, USA) and SPSS version 21 (SPSS, Chicago, IL, USA). Normal distribution of the data was confirmed using the Shapiro–Wilk test. Continuous data are reported as median (interquartile range). Continuous variables were compared between each time point using repeated measures analysis of variance with *post-hoc* Tukey-Kramer honestly significant difference test. Partial correlation analysis controlling for the effect of dogs was used to determine the relationship between hemodynamic variables and echocardiographic indices. Partial correlation analysis was developed with echocardiographic indices as an outcome variable and hemodynamic variables as explanatory variables. Dogs were treated as a categorical factor using a dummy variable with five degrees of freedom. Subsequently, to determine the independent predictive value of mean PAP, PVR, and CI, multiple linear regression analysis with forward stepwise selection and Akaike information criteria was performed with the *P* levels for entry from the model set at <0.20. Candidate predictors were mitral E wave, systolic EI, diastolic EI, S'_TV_, tricuspid annular plane systolic excursion, FAC, RV Tei index, free wall RVLS, septal RVLS, and RV-SD6. *P*-values < 0.05 were considered statistically significant.

## Results

### Changes in Hemodynamic Variables

**Figure 2:**
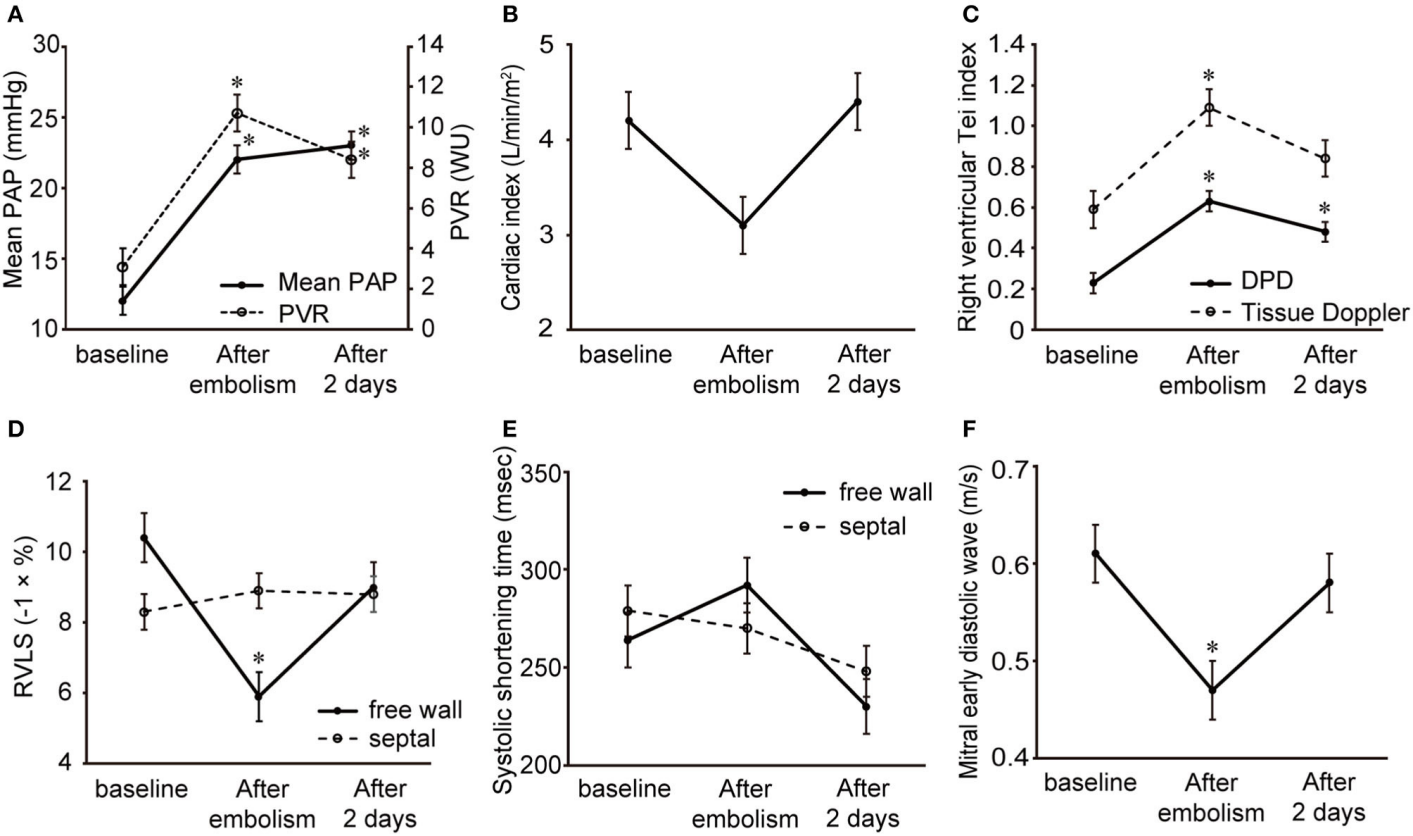
Changes in the hemodynamic variables and echocardiographic indices in a dog model of acute pulmonary embolism. Data are shown as the mean ± standard deviation. **(A)** Mean pulmonary arterial pressure and pulmonary vascular resistance. **(B)** Cardiac index. **(C)** Right ventricular Tei index by dual pulsed-wave Doppler and tissue Doppler. **(D)** Free wall and septal right ventricular longitudinal strain. **(E)** Systolic shortening time of free wall and septum. **(F)** Mitral early diastolic wave. *P < 0.05 compared with the baseline.

Changes in the hemodynamic variables in a dog model of acute pulmonary embolism are summarized in Table 1 and Figures 2A,B. Systolic PAP, diastolic PAP, mean PAP, mean RV pressure, mean central venous pressure, and PVR were significantly higher than baseline values during the acute period. The mean arterial blood pressure significantly decreased compared to those at baseline during the acute period. However, systolic PAP, diastolic PAP, mean PAP, and PVR remained higher during the compensation period than at baseline. The max dP/dt was significantly increased during the compensation period. In contrast, the mean RV pressure, mean central venous pressure, and CI during the compensation period did not differ from the baseline values.

**Table 1:** Comparison of hemodynamic variables at baseline, acute period, and compensation period in a dog model of acute pulmonary embolism.

	**Baseline**	**Acute period**		**Compensation period**	
	**value**	**value**	***P***	**value**	***P***
Heart rate (beat/min)	110 (101–117)	102 (95–111)	0.51	106 (103–111)	0.92
Mean BP (mmHg)	58 (56–59)	47 (44–54)*	0.006	51 (48–58)	0.19
Systolic PAP (mmHg)	15 (14–18)	26 (23–27)*	<0.001	26 (24–29) *	<0.001
Mean PAP (mmHg)	12 (10–14)	23 (21–24)*	<0.001	24 (20–25)*	<0.001
Diastolic PAP (mmHg)	10 (7–11)	19 (17–20)*	<0.001	20 (15–21)*	<0.001
Mean RVP (mmHg)	9 (7–11)	14 (12–17)*	0.019	13 (10–13)	0.28
Mean CVP (mmHg)	2 (1–2)	4 (3–6)*	0.016	2 (1–3)	0.98
Mean PAWP (mmHg)	6 (4–6)	5 (4–7)	0.81	5 (2–7)	0.81
CI (L/min/m^2^)	4.4 (3.5–4.4)	2.8 (2.6–3.7)	0.085	4.1 (3.6–5.2)	0.90
Max dP/dt (mmHg/sec)	117 (107–148)	173 (141–195)	0.16	201 (146–255)*	0.016
PVR (WU)	3.1 (2.9–3.4)	10.5 (7.8–13.4)*	<0.001	8.5 (6.1–9.9)*	0.002
SVR (WU)	26.1 (22.1–29.9)	26.1 (23.1–30.5)	0.43	24.9 (20.9–26.4)	0.62
Rp/Rs	0.12 (0.11–0.14)	0.36 (0.33–0.50)*	<0.001	0.36 (0.30–0.41)*	<0.001

Continuous variables were displayed as median (interquartile range). BP, Blood pressure; PAP, Pulmonary arterial pressure; RVP, Right ventricular pressure; CVP, Central venous pressure; PAWP, Pulmonary artery wedge pressure; CI, Cardiac index; Max dP/dt, Maximum positive rate of RV pressure change; PVR, Pulmonary vascular resistance; SVR, Systemic vascular resistance; Rp/Rs, PVR to SVR ratio. *P < 0.05 compared with the baseline.

### Changes in Echocardiographic Indices

**Figure 3:**
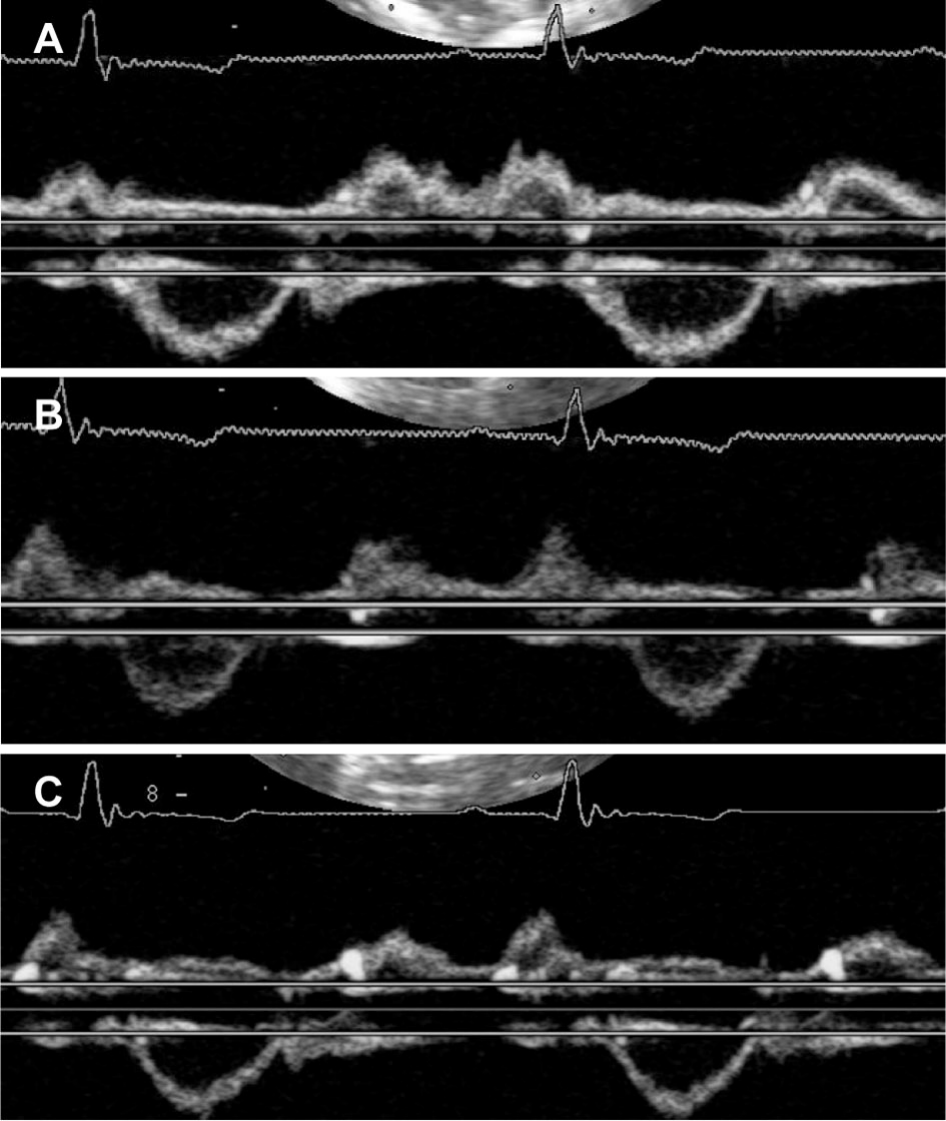
Representative images of right ventricular Tei index by dual pulsed-wave Doppler at baseline **(A)** acute period **(B)** and compensation period **(C)**. **(A)** Right ventricular Tei index was 0.27 at baseline. **(B)** In acute period, right ventricular Tei index was increased (0.77). **(C)** In compensation period, right ventricular Tei index was still higher than baseline (0.63).

**Figure 4:**
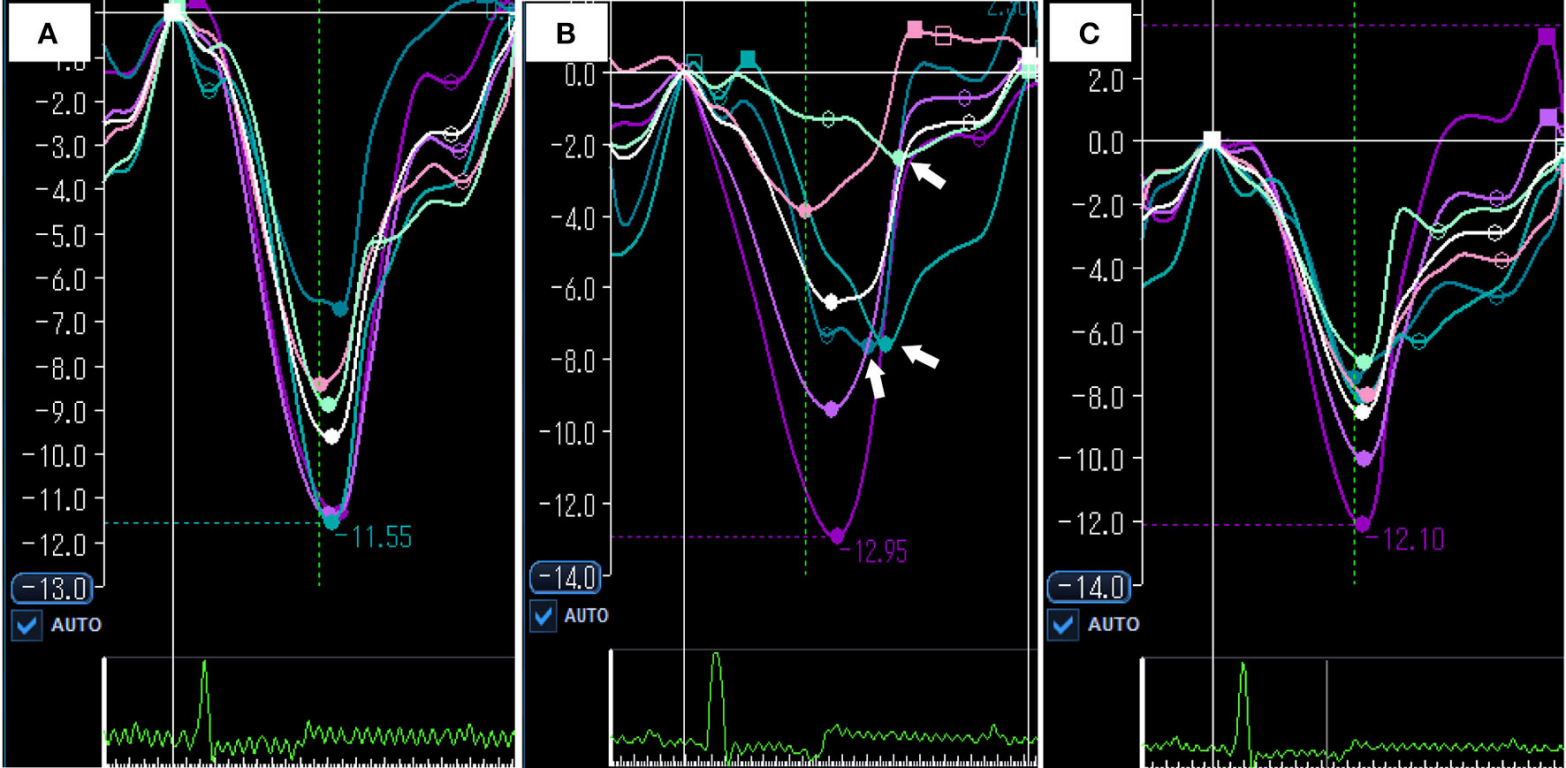
Representative images of speckle tracking echocardiography at baseline **(A)** acute period **(B)** and compensation period **(C)**. **(A)** Global RVLS was −9.6% and RV-SD6 was 16.8 msec at baseline. **(B)** In acute period, global RVLS was significantly impaired compared with baseline (– 6.4%). Systolic shortening times of free wall were delayed (white arrows), and RV-SD6 was increased (81.5 msec). **(C)** In compensation period, global RVLS and RV-SD6 were returned to the baseline level (−8.6% and 10.1 msec). White color curve indicates global RVLS. RVLS, right ventricular longitudinal strain; RV-SD6, standard deviation of systolic shortening time of right ventricular six segments.

The echocardiographic index changes in a dog model of acute pulmonary embolism are summarized in Table 2 and Figures 2C–F. Examples of baseline, acute, and compensation periods are shown in Figures 3, 4, respectively.

**Table 2:** Comparison of echocardiographic indices at baseline, acute period, and compensation period in a dog model of acute pulmonary embolism.

	**Baseline**	**Acute period**		**Compensation period**	
	**value**	**value**	***P***	**value**	***P***
LVIDDN	1.62 (1.44–1.67)	1.42 (1.29–1.58)	0.21	1.58 (1.45–1.66)	0.98
Systolic EI	1.03 (1.01–1.08)	1.26 (1.09–1.36)*	0.013	1.10 (1.04–1.18)	0.44
Diastolic EI	1.07 (1.04–1.11)	1.30 (1.25–1.38)*	<0.001	1.14 (1.11–1.20)	0.28
Mitral E (m/s)	0.60 (0.57–0.65)	0.44 (0.39–0.57)*	0.027	0.59 (0.54–0.62)	0.87
Mitral A (m/s)	0.38 (0.30–0.42)	0.29 (0.26–0.38)	0.32	0.35 (0.29–0.40)	0.78
S'_TV_ (cm/sec)	5.7 (4.9–6.7)	5.0 (4.7–6.1)	0.74	5.3 (4.5–6.1)	1.0
TAPSE (mm)	7.6 (6.1–8.7)	4.9 (3.6–6.4)	0.085	5.6 (4.3–7.8)	0.80
RVEDA (cm^2^)	9.5 (8.8–10.3)	11.0 (9.8–12.2)	0.073	9.4 (8.6–10.3)	0.99
RVESA (cm^2^)	7.5 (6.6–7.9)	9.2 (8.3–10.4)*	0.009	7.5 (6.2–8.1)	0.99
FAC (%)	21.8 (21.5–28.2)	15.3 (12.5–17.8)*	0.005	22.4 (20.5–27.6)	0.91
Tei index (DPD)	0.24 (0.18–0.27)	0.66 (0.49–0.79)*	<0.001	0.49 (0.37–0.56)*	0.008
Tei index (TDI)	0.58 (0.48–0.70)	1.11 (0.86–1.25)*	0.004	0.86 (0.67–1.04)	0.15
Global RVLS (- %)	9.3 (8.5–10.0)	7.2 (5.8–8.0)*	0.006	8.9 (8.0–9.6)	0.72
Free wall RVLS (- %)	10.3 (9.1–11.7)	6.4 (4.5–6.9)*	<0.001	9.6 (7.2–10.7)	0.34
Basal free wall	13.7 (9.4–14.7)	6.7 (4.0–9.7)*	0.033	10.8 (8.9–14.4)	0.75
Middle free wall	12.6 (9.1–13.4)	8.2 (5.3–9.9)*	0.041	10.7 (7.8–12.1)	0.53
Apical free wall	6.0 (4.5–10.0)	3.6 (2.3–6.4)	0.12	6.0 (5.1–8.1)	0.90
Septal RVLS (- %)	8.6 (7.5–9.0)	8.8 (8.2–9.3)	0.69	9.4 (8.3–9.7)	1.0
Basal septum	9.6 (8.7–11.2)	12.8 (10.9–13.1)	0.083	11.5 (9.9–12.5)	0.55
Middle septum	9.6 (7.2–10.3)	10.0 (8.2–10.6)	0.59	10.1 (8.9–10.8)	0.97
Apical septum	5.8 (3.9–7.8)	5.3 (3.2–6.1)	0.25	7.0 (5.7–8.0)	0.80
Free wall SST (msec)	261 (245–291)	301 (257–328)	0.37	241 (204–245)	0.22
Basal free wall	284 (218–296)	325 (263–339)*	0.028	230 (206–255)	0.46
Middle free wall	261 (247–284)	333 (258–355)*	0.002	244 (206–255)	0.39
Apical free wall	264 (249–284)	315 (264–380)*	0.049	252 (235–263)	0.83
Septal SST (msec)	278 (257–311)	274 (232–306)	0.25	254 (224–268)	0.25
Basal septum	281 (244–292)	270 (223–292)	0.62	250 (229–286)	0.89
Middle septum	277 (246–313)	269 (227–288)	0.32	256 (224–271)	0.75
Apical septum	275 (238–304)	298 (260–340)	0.21	256 (222–278)	0.66
Free wall delay (msec)	−15 (–24–5)	5 (–15–81)	0.15	– 11 (–41–0)	1.0
RV-SD6 (msec)	23 (19–28)	69 (31–81)*	0.013	22 (13–36)	0.99

Continuous variables were displayed as median (interquartile range). LVIDDN, normalized left ventricular internal diameter in diastole; EI, Eccentricity index; E, Early diastolic wave; A, Late diastolic wave; S'_TV_, Systolic tricuspid annular velocity; TAPSE, Tricuspid annular plane systolic excursion; RVEDA, Right ventricular end-diastolic area; RVESA, Right ventricular end-systolic area; FAC, Fractional area change; DPD, Dual pulsed-wave Doppler; TDI, Tissue Doppler; RVLS, Right ventricular longitudinal strain; SST, Systolic shortening time; RV-SD6, Standard deviation of the time to peak longitudinal strain of six right ventricular segments. *P < 0.05 compared with the baseline.

Mitral E waves were significantly decreased in the acute period and returned to baseline levels during the compensation period. Systolic and diastolic EI increased significantly in the acute period and returned to baseline levels during the compensation period. The right ventricle was significantly dilated and several RV function indices, including FAC, tricuspid annular plane systolic excursion, and RV Tei index by TDI decreased in the acute period and recovered to baseline levels during the compensation period. Unlike other echocardiographic indices, the RV Tei index by DPD was significantly increased after embolism and remained elevated 2 days later.

**Figure 5:**
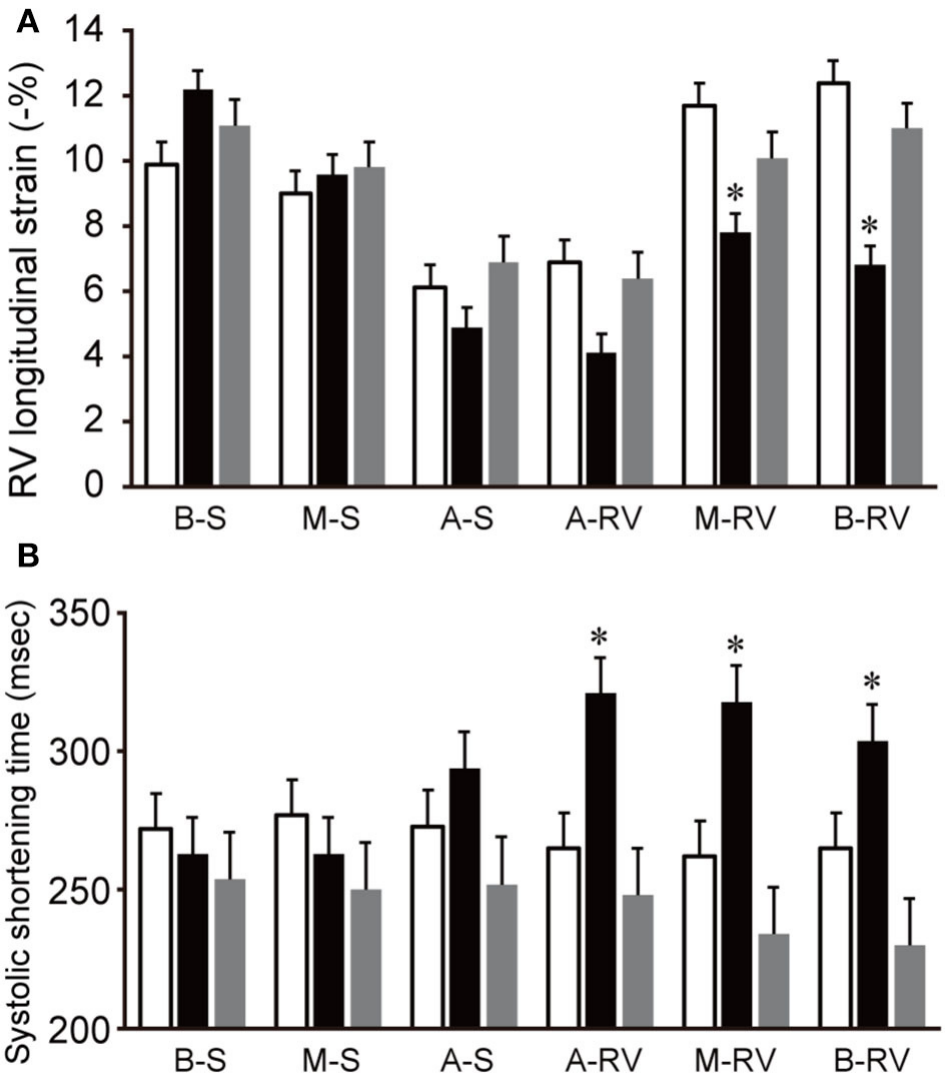
Changes in segmental RVLS and SST in a dog model of acute pulmonary embolism. Data are shown as the mean ± standard deviation. **(A)** Basal and middle free wall RVLS were significantly lower than baseline in acute period, and were return to the baseline level in compensation period. **(B)** All segmental free wall SST were significant long compared with baseline in acute period. White bar, baseline; black bar, acute period; gray bar, compensation period. A-RV, apical free wall; A-S, apical septum; B-RV, basal free wall; B-S, basal septum; M-RV, middle free wall; M-S, middle septum. RVLS, right ventricular longitudinal strain; SST, systolic shortening time. *P < 0.05 compared with the baseline.

Free wall and global RVLS were significantly reduced in the acute period and returned to baseline levels during the compensation period. In contrast, septal RVLS did not change in a dog model of acute pulmonary embolism. Figure 5 shows the changes in segmental RVLS and SST in a dog model of acute pulmonary embolism. Basal and middle free wall RVLS were significantly decreased in the acute period. All segmental RVLS during the compensation period did not differ from the baseline values. All segmental free wall SST was significantly longer than baseline in the acute period, resulting in increased RV-SD6.

### Partial Correlation Analysis

Partial correlations controlling for the effect in dogs between echocardiographic indices and hemodynamic variables are summarized in Table 3. Free-wall segmental RVLS was negatively correlated with PVR. In contrast, basal septal RVLS was positively correlated with PVR. Mitral E wave was positively correlated with CI (r = 0.85) and negatively correlated with PVR, RV-SD6, and diastolic and systolic EI (r = −0.76; r = −0.70; r = −0.76: r = −0.80, respectively).

**Table 3:** Partial correlation analysis of echocardiographic indices and hemodynamic variables controlling for the effect of dogs in a dog model of acute pulmonary embolism.

**Variable**	**Mean PAP**		**PVR**		**CI**	
	***r***	***P***	***r***	***P***	***r***	***P***
Mitral E wave	−0.43^*^	0.078	−0.76^*^	<0.001	0.85^*^	<0.001
Systolic EI	0.57^*^	0.013	0.84^*^	<0.001	– 0.70^*^	0.002
Diastolic EI	0.55^*^	0.017	0.79^*^	<0.001	−0.62^*^	0.006
S'_TV_	−0.10^*^	0.70	−0.38^*^	0.12	0.65^*^	0.004
TAPSE	−0.51^*^	0.032	−0.52^*^	0.026	0.31^*^	0.21
FAC	−0.20^*^	0.42	−0.42^*^	0.079	0.48^*^	0.042
Tei index (DPD)	0.82^*^	<0.001	0.91^*^	<0.001	−0.50^*^	0.036
Tei index (TDI)	0.59^*^	0.010	0.70^*^	0.001	−0.54^*^	0.022
Free wall RVLS	0.62^*^	0.006	0.77^*^	<0.001	−0.66^*^	0.003
Septal RVLS	0.15^*^	0.56	0.20^*^	0.43	−0.25^*^	0.31
RV-SD6	0.35^*^	0.15	0.61^*^	0.007	−0.55^*^	0.018

* *P < 0.05*.

### Multiple Regression Analysis

Multiple linear regression analysis with all candidate predictors (model 1) revealed that the RV Tei index by DPD was an independent predictor of the mean PAP (β = 0.88, *P* < 0.001) and PVR (β = 0.99, *P* < 0.001). The mitral E wave was an independent predictor of CI (β = −0.77, *P* = 0.001).

In a subanalysis model without the RV Tei index by DPD (model 2), the RV Tei index by TDI was an independent predictor of mean PAP (β = 0.76, *P* = 0.006). Multiple linear regression analysis (model 2) revealed that the RV Tei index by TDI and systolic EI were independent predictors of PVR (RV Tei index by TDI, β = 0.55, *P* = 0.01; systolic EI, β = 0.56, *P* = 0.01).

## Discussion

This is the first study to validate the relationship between echocardiographic indices and hemodynamics measured by right heart catheterization in a dog model of acute pulmonary embolism during the acute and compensation periods. The data on immediately after pulmonary embolism and compensation period have not reported to date. The major findings of our study are as follows: (1) low blood pressure, RV dilation, LV compression, septal flattening, impaired echocardiographic indices of RV function, and RV dyssynchrony were induced by acute pulmonary embolism, despite only mild elevation in PAP during the acute period; (2) blood pressure and echocardiographic indices, including mitral E wave, FAC, free wall RVLS, and RV-SD6, were reversed, PAP and RV Tei index by DPD did not normalize during the compensation period; (3) RV Tei index was an independent predictor of RV afterload and mitral E wave was an independent predictor of CI.

Microsphere injection caused an acute increase in RV afterload, resulting in a reduction in blood pressure. Although this dog model exhibited only mild elevation in mean PAP (<25 mmHg), severe RV dilation, RV dysfunction, and hemodynamic instability (low blood pressure) occurred. These changes were consistent with the fact that the RV is vulnerable to acute pressure overload.

### Acute Period

In the present study, the RV Tei index, a systolic and diastolic function index, measured by DPD and TDI, increased significantly after pulmonary embolism. Sugiura et al. and Ichikawa et al. demonstrated that the RV Tei index was impaired in acute PTE patients with reduced stroke volume (9, 11). Our results are in line with those reported by Sugiura and Ichikawa. In addition, our previous study revealed that the RV Tei index by DPD was significantly increased in dog models with mild RV pressure overload (12). The impaired RV Tei index reflects an acute increase in the RV afterload.

Acute RV pressure overload significantly reduced basal and middle free wall RVLS in a dog model of acute pulmonary embolism, which resulted in the impairment of free wall RVLS. Our results are consistent with those in human patients with acute PTE (10). Our previous study also demonstrated that acute mild RV pressure overload significantly reduced the basal and middle RVLS in dogs (12). Based on these findings, the RV free wall, particularly in the basal and middle segments, was sensitive to acute RV pressure overload in dogs. In the present study, apical free wall RVLS did not change significantly after acute pulmonary embolism, similar to the results in human patients with acute PTE and dog models of mild RV pressure overload (10, 12). In contrast, several human clinical observations demonstrated that patients with acute PTE had reduced RVLS of all free wall segments, including the apical free wall (5, 8, 9, 11). Although addressing the underlying factors responsible for this difference in apical free wall RVLS would be difficult, the severity or duration of RV pressure overload may be related to this difference. Systolic PAP was 25 mmHg (95% CI, 22–28 mmHg), and the duration of RV pressure overload was 15 min in the present study. In human clinical observations, while the accurate duration of RV pressure overload was unknown, systolic PAP was higher than that in the present study. Severe acute RV pressure overload can cause impairment of apical free wall RVLS.

In contrast to free wall RVLS, septal RVLS did not change during the acute period. A previous study demonstrated similar findings using dog models of acute mild RV pressure overload (12). Based on these findings, the interventricular septum may be less sensitive to acute RV pressure overload in acute pulmonary embolism. Therefore, in a clinical setting, the measurement of septal RVLS may not be useful for assessing RV function in dogs with acute PTE.

Right ventricular dyssynchrony determined by RV-SD6 occurred after acute pulmonary embolism in the present study. This finding agrees with clinical observations in acute PTE in humans (9, 11) and a dog with acute PTE (19), as well as a study using dog models of acute RV pressure overload (12). Moreover, similar to studies in humans with acute PTE and dog models of acute RV pressure overload (9, 12), free wall systolic delay was the factor causing RV dyssynchrony in acute pulmonary embolism. The present findings support the premise that acute RV pressure overload causes free wall systolic delay, resulting in RV dyssynchrony in dogs.

### Compensation Period

In a dog model of acute pulmonary embolism, free wall RVLS and RV-SD6 returned to normal values during the compensation period despite persistently high PAP and PVR. After embolism, RV pulmonary arterial uncoupling (RV systolic function maladaptation to afterload) may occur, resulting in low blood pressure. Max dP/dt significantly increased and a high RV afterload persisted during the compensation period, suggesting that RV-pulmonary arterial coupling improved. This finding suggests that acute RV pressure overload and RV pulmonary arterial uncoupling may contribute to RV dysfunction and dyssynchrony. However, in our study, the gold standard indices of RV systolic function and afterload, such as ventricular end-systolic elastance and effective arterial elastance, were not measured. Further studies with RV pressure-volume loops are required to clarify this point.

Regarding the reversibility of RV function, conflicting findings have been reported in human patients with acute PTE (7–9). One clinical observation demonstrated that human patients with acute PTE had lower basal and middle free wall RVLS after the elimination of acute RV pressure overload than healthy controls (9). Conversely, other human studies (7, 8) revealed the reversibility of RVLS after treatment in patients with acute PTE, similar to our study. It is difficult to address this discrepancy because the severity of RV pressure overload and the timing of echocardiography after treatment in these studies were not uniform.

The reversibility of RV dyssynchrony has been addressed in a human study (9). After treatment, SST in the middle and apical free wall was significantly shortened, resulting in the restoration of RV synchronicity in patients with acute PTE (9). In the present study, SST in all six segments tended to be shortened during the compensation period compared with those at baseline. Although the mechanisms underlying the shortened SST during the compensation period are unclear, they could compensate for the non-uniformity of RV myocardial contraction after acute pulmonary embolism.

### Diagnostic utility in acute pulmonary embolism

In the clinical setting, dogs with acute PTE may be examined during both acute and compensatory periods. Based on the present results, if a dog with acute PTE is examined by echocardiography during the compensation period, many echocardiographic indices would not be detected as abnormal regardless of persistently elevated PAP and PVR. Echocardiographic indices that can be detected as abnormal in each period are ideal in the clinical setting. In contrast to other echocardiographic indices, the RV Tei index was impaired in both the acute and compensation periods. In human patients with acute PTE, the RV Tei index recovered after the elimination of RV pressure overload, but it was still impaired compared with that in healthy subjects (8). In addition, the RV Tei index by DPD was significantly correlated with mean PAP and PVR, and it was an independent predictor of mean PAP and PVR in a dog model of acute pulmonary embolism, similar to human patients with acute PTE (11). Our results indicate that the RV Tei index by DPD is the most useful echocardiographic index for the diagnosis of acute PTE in dogs. Measurement of the RV Tei index by DPD may be promising because right heart catheterization is difficult to perform in a clinical setting. One limitation of the RV Tei index by DPD is the limited availability of ultrasonographic systems with DPD echocardiography. For this reason, we also applied the RV Tei index by TDI, which is a more commonly used application in dogs. The RV Tei index by TDI was an independent predictor of mean PAP and PVR in a multiple linear regression (model 2) without the RV Tei index by DPD. This finding suggests that the RV Tei index by TDI is also useful for diagnosing acute PTE when DPD echocardiography is not available.

A simple echocardiographic index is required in clinical settings because dogs with acute PTE are likely to have respiratory distress or hemodynamic instability. In the present study, the mitral E wave was an independent predictor of CI. This finding indicates that a low mitral E wave is an important factor causing low cardiac output in acute pulmonary embolism. Mitral E wave may be useful for assessing cardiac output in dogs with acute PTE because of the convenience of measurement using a pulsed-wave Doppler. While a low mitral E wave or E/A was observed in human patients with acute PTE (11, 20), the association between mitral E wave and cardiac output has not been addressed to date. Other researchers reported that a low mitral E wave was associated with cardiac events in human patients with chronic PH (21). A low mitral E-wave may be related to direct or sequential ventricular interdependence. In direct interdependence, leftward interventricular septal bowing compresses the LV cavity, resulting in impaired LV diastolic function and decreased LV preload and cardiac output (22, 23). Sequential interdependence is caused by a decrease in RV output due to increased afterload or RV dysfunction, resulting in decreased LV preload and low cardiac output (21, 24). In the present study, left septal bowing, increased RV afterload, and RV dysfunction occurred in dogs with acute pulmonary embolism. In addition, mitral E wave was significantly correlated with EI, PVR, RV Tei index, and free wall RVLS. These findings suggest that direct and sequential ventricular interdependencies occur in dogs with acute PTE and cause low cardiac output. Additionally, RV dyssynchrony impaired LV filling due to delayed systolic peak in the RV during LV early diastole and decreased RV output (23, 25). Since mitral E wave was significantly correlated with RV-SD6 in a dog model of acute pulmonary embolism, RV dyssynchrony could cause ventricular interdependence. However, there is a limitation of the mitral E wave. According to a previous paper, transmitral flow is affected by age, and mitral E wave decreases in dogs older than 6 years (26). Therefore, care should be taken when interpreting the results of mitral E waves in older dogs (>6 years).

### Limitations

The present study had several limitations. First, only a small number of dogs were included in the present study. Therefore, a type 2 error can occur. Although several variables did not change in the present study, the small sample size could have rendered the study underpowered to detect actual differences. Second, acute pulmonary embolism caused only a mild increase in the mean PAP (<25 mmHg). Caution must be exercised when extrapolating these data to dogs with severe acute PTE. Third, we cannot exclude the effects of anesthesia on hemodynamics, myocardial function, and echocardiographic indices. Fourth, in the present study, we measured max dP/dt as the RV systolic function index, but it does not reflect accurate RV systolic function because of load dependence. Further studies measuring load-independent indices derived from RV pressure-volume loops, such as ventricular end-systolic elastance, are required to clarify the association between echocardiographic indices and RV systolic function.

## Conclusions

Several echocardiographic indices of RV function, such as free wall RVLS and RV-SD6, were impaired after acute pulmonary embolism and recovered after 2 days. In contrast, the RV Tei index was not only impaired during the acute period after pulmonary embolism, but the impairment also persisted after 2 days and was an independent predictor of RV afterload in multiple regression analysis. Therefore, the RV Tei index may be useful for the diagnosis of acute PTE in this model, but not in dogs with clinical PTE.

## Data Availability Statement

The original contributions presented in the study are included in the article/supplementary material, further inquiries can be directed to the corresponding author.

## Ethics Statement

The animal study was reviewed and approved by the Laboratory Animal Experimentation Committee of the Graduate School of Veterinary Medicine, Hokkaido University (Approval No. 15-0087).

## Author Contributions

TM and KN designed the study. TM wrote the manuscript. KN performed echocardiography. TM and TO analyzed the data. TM, KN, TO, SK, and SM performed cardiac catheterization. KN and MT edited the manuscript and approved the final version. All authors contributed to the articles and approved the final manuscript.

## Funding

This study was partially supported by a Grant-in-Aid for Scientific Research from the Japanese Society for the Promotion of Science (No. 19K06422).

## Conflict of Interest

The authors declare that the research was conducted in the absence of any commercial or financial relationships that could be construed as a potential conflict of interest.

## Publisher's Note

All claims expressed in this article are solely those of the authors and do not necessarily represent those of their affiliated organizations, or those of the publisher, the editors and the reviewers. Any product that may be evaluated in this article, or claim that may be made by its manufacturer, is not guaranteed or endorsed by the publisher.

## References

[1] Macnee W. Pathophysiology of cor pulmonale in chronic obstructive pulmonary disease. Am J Respir Crit Care Med. (1994) 150:833–52. 10.1164/ajrccm.150.3.8087359 8087359

[2] Johnson LR Lappin MR Baker DC. Pulmonary thromboembolism in 29 dogs: 1985–1995. J Vet Intern Med. (1999) 13:338–45. 10.1111/j.1939-1676.1999.tb02192.x 10449226

[3] Stein PD Fowler SE Goodman LR Gottschald A Hales CA Hull RD. Multidetector computed tomography for acute pulmonary embolism. N Engl J Med. (2006) 354:2317–27. 10.1056/NEJMoa052367 16738268

[4] Goggs R Chan DL Benigni L Hirst C Kellett-Gregory L Fuentes VL. Comparison of computed tomography pulmonary angiography and point-of-care tests for pulmonary thromboembolism diagnosis in dogs. J Small Anim Pract. (2014) 55:190–7. 10.1111/jsap.12185 24521253 PMC4477636

[5] Platz E Hassanein AH Shah A Goldhaber SZ Solomon SD. Regional right ventricular strain pattern in patients with acute pulmonary embolism. Echocardiography. (2012) 29:464–70. 10.1111/j.1540-8175.2011.01617.x 22276918

[6] Wright L Dwyer N Power J Kritharides L Celermajer D Marwick TH. Right ventricular systolic function responses to acute and chronic pulmonary hypertension: assessment with myocardial deformation. J Am Soc Echocardiogr. (2016) 29:259–66. 10.1016/j.echo.2015.11.010 26944627

[7] Park JH Park YS Park SJ Lee JH Choi SW Jeong JO. Midventricular peak systolic strain and Tei index of the right ventricle correlated with decreased right ventricular systolic function in patients with acute pulmonary thromboembolism. Int J Cardiol. (2008) 125:319–24. 10.1016/j.ijcard.2007.02.030 17434620

[8] Vitarelli A Barillà F Capotosto L D'Angeli I Truscelli G De Maio M. Right ventricular function in acute pulmonary embolism: A combined assessment by three-dimensional and speckle-tracking echocardiography. J Am Soc Echocardiogr. (2014) 27:329–38. 10.1016/j.echo.2013.11.013 24325961

[9] Sugiura E Dohi K Onishi K Takamura T Tsuji A Ota S. Reversible right ventricular regional non-uniformity quantified by speckle-tracking strain imaging in patients with acute pulmonary thromboembolism. J Am Soc Echocardiogr. (2009) 22:1353–9. 10.1016/j.echo.2009.09.005 19836202

[10] Ramberg E Olausson M Jørgensen TBS Nepper ML Bhardwaj P Binko TS. Right atrial and ventricular function evaluated with speckle tracking in patients with acute pulmonary embolism. Am J Emerg Med. (2017) 35:136–43. 10.1016/j.ajem.2016.09.059 27780650

[11] Ichikawa K Dohi K Sugiura E Sugimoto T Takamura T Ogihara Y. Ventricular function and dyssynchrony quantified by speckle-tracking echocardiography in patients with acute and chronic right ventricular pressure overload. J Am Soc Echocardiogr. (2013) 26:483–92. 10.1016/j.echo.2013.02.010 23528714

[12] Morita T Nakamura K Osuga T Yokoyama N Morishita K Sasaki N. Changes in right ventricular function assessed by echocardiography in dog models of mild RV pressure overload. Echocardiography. (2017) 34:1040–9. 10.1111/echo.13560 28493465

[13] Morita T Nakamura K Osuga T Kawamoto S Miki S Sasaoka K. Acute effects of intravenous pimobendan administration in dog models of chronic precapillary pulmonary hypertension. J Vet Cardiol. (2020) 32:16–27. 10.1016/j.jvc.2020.09.003 33080489

[14] Tai TC Huang HP. Echocardiographic assessment of right heart indices in dogs with elevated pulmonary artery pressure associated with chronic respiratory disorders, heartworm disease, and chronic degenerative mitral valvular disease. Vet Med. (2013) 58:613–20. 10.17221/7180-VETMED

[15] Morita T Nakamura K Osuga T Lim SY Yokoyama N Morishita K. Repeatability and reproducibility of right ventricular Tei index valves derived from three echocardiographic methods for evaluation of cardiac function in dogs. Am J Vet Res. (2016) 77:715–20. 10.2460/ajvr.77.7.715 27347824

[16] Hori Y Kunihiro S Hoshi F Higuchi S. Comparison of the myocardial performance echocardiography and tissue doppler imaging. Am J Vet Res. (2007) 68:1177–82. 10.2460/ajvr.68.11.1177 17975971

[17] Morita T Nakamura K Osuga T Yokoyama N Nisa K Morishita K. The repeatability and characteristics of right ventricular longitudinal strain imaging by speckle tracking echocardiography in healthy dogs. J Vet Cardiol. (2017) 19:351–62. 10.1016/j.jvc.2017.05.001 28739084

[18] Bazett HC. An analysis of the time-relations of electrocardiograms. Heart. (1920) 7:353–70.

[19] Morita T Nakamura K Osuga T Hanazono K Morishita K Takiguchi M. Change in right ventricular function in an American cocker spaniel with acute pulmonary thromboembolism. J Vet Med Sci. (2019) 81:1259–65. 10.1292/jvms.19-0082 31292347 PMC6785625

[20] Hsiao SH Lee CY Chang SM Yang SH Lin SK Huang WC. Pulmonary embolism and right heart function: Insights from myocardial Doppler tissue imaging. J Am Soc Echocardiogr. (2006) 19:822–8. 10.1016/j.echo.2006.01.011 16762763

[21] Motoji Y Tanaka H Fukuda Y Sano H Ryo K Imanishi J. Interdependence of right ventricular systolic function and left ventricular filling and its association with outcome for patients with pulmonary hypertension. Int J Cardiovasc Imaging. (2015) 31:691–8. 10.1007/s10554-015-0598-x 25614330

[22] Gan CTJ Lankhaar JW Marcus JT Westerhof N Marques KM Bronzwaer JGF. Impaired left ventricular filling due to right-to-left ventricular interaction in patients with pulmonary arterial hypertension. Am J Physiol - Hear Circ Physiol. (2006) 290:1528–33. 10.1152/ajpheart.01031.2005 16284226

[23] Burkett DA Slorach C Patel SS Redington AN Ivy DD Mertens L. Impact of pulmonary hemodynamics and ventricular interdependence on left ventricular diastolic function in children with pulmonary hypertension. Circ Cardiovasc Imaging. (2016) 9:1–11. 10.1161/CIRCIMAGING.116.004612 27581953 PMC5012318

[24] Gurudevan S V Malouf PJ Auger WR Waltman TJ Madani M Raisinghani AB. Abnormal left ventricular diastolic filling in chronic thromboembolic pulmonary hypertension. True diastolic dysfunction or left ventricular underfilling? J Am Coll Cardiol. (2007) 49:1334–9. 10.1016/j.jacc.2007.01.028 17394966

[25] Marcus JT Gan CT Zwanenburg JJ Boonstra A Allaart CP Götte MJ. Interventricular mechanical asynchrony in pulmonary arterial hypertension: left-to-right delay in peak shortening is related to right ventricular overload and left ventricular underfilling. J Am Coll Cardiol. (2008) 51:750–7. 10.1016/j.jacc.2007.10.041 18279740

[26] Schober KE Fuentes VL. Effects of age, body weight, and heart rate on transmitral and pulmonary venous flow in clinically normal dogs. Am J Vet Res. (2001) 62:1447–54. 10.2460/ajvr.2001.62.1447 11560276

